# Mycobacterium tuberculosis modulates NUDT21-mediated alternative polyadenylation to enhance FTH1 expression in macrophages and promotes intracellular growth

**DOI:** 10.3389/fcimb.2025.1578163

**Published:** 2025-05-02

**Authors:** Xiaoqian Liu, Ningjian Cai, Youchao Dai, Xinchun Chen, Xiaobin Zeng

**Affiliations:** ^1^ College of Life Science and Technology, Wuhan Polytechnic University, Wuhan, Hubei, China; ^2^ Guangdong Provincial Key Laboratory of Infection Immunity and Inflammation, Department of Pathogen Biology, Shenzhen University Medical School, Shenzhen, China; ^3^ Guangzhou Medical Research Institute of Infectious Diseases, Infectious Disease Center, Guangzhou Eighth People’s Hospital, Guangzhou Medical University, Guangzhou, Guangdong, China; ^4^ Center Lab of Longhua Branch and Department of Infectious Disease, Shenzhen People’s Hospital (The Second Clinical Medical College, Jinan University, The First Affiliated Hospital, Southern University of Science and Technology), Shenzhen, Guangdong, China

**Keywords:** *Mycobacterium tuberculosis*, alternative polyadenylation, *FTH1*, macrophage, NUDT21

## Abstract

Ferritin heavy chain 1 (FTH1) is a key iron-storage protein that regulates iron availability, supports immune defense, and prevents iron-induced toxicity. During *Mycobacterium tuberculosis* (*Mtb*) infection, macrophages enhance FTH1 expression to sequestrate iron and limit *Mtb* growth. However, *Mtb* can exploit the host ferritinophagy pathway to degrade FTH1 and release iron, thereby promoting its survival. Although FTH1 plays an essential role in host–pathogen interaction during *Mtb* infection, its regulation remains unclear. Previous studies suggest that post-transcriptional mechanism, particularly alternative polyadenylation (APA), are critical in immune responses. We propose that APA, which determines the length of a transcript’s 3′UTR, may regulate *FTH1* expression during *Mtb* infection. Our study demonstrates that *Mtb* induces APA of *FTH1* in macrophages, favoring the production of longer isoforms that enhance protein synthesis. Mechanistically, *Mtb* disrupts the interaction between NUDT21 and CPSF6, impairing NUDT21’s ability to bind UGUA motifs in the *FTH1* 3′UTR, a key step in polyadenylation site selection. Silencing NUDT21 reduces macrophage bactericidal activity against *Mtb*, highlighting its role in immune defense. These findings reveal a novel *Mtb*-driven mechanism that enhances *FTH1* expression via the NUDT21-mediated APA pathway in macrophages, suggesting that *Mtb* manipulates this process to promote its survival. This study provides new insights into tuberculosis pathogenesis and points to potential avenues for therapeutic exploration.

## Introduction

Tuberculosis (TB), caused by *Mycobacterium tuberculosis* (*Mtb*), remains one of the world’s most significant infectious diseases (World Health Organization, 2024). Macrophages, key players in the innate immune system, have a paradoxical role in TB pathogenesis (Bo et al., 2023; Weiss and Schaible, 2015). While they can restrict *Mtb* growth through bactericidal mechanisms, they may also support bacterial persistence by providing a protective niche (Hmama et al., 2015; Pisu et al., 2020; Kurthkoti et al., 2017). Central to macrophage defense against *Mtb* is iron homeostasis, which supports processes such as oxidative killing, antigen presentation, and phagocytosis (Ganz and Nemeth, 2015). However, excessive intracellular iron can induce oxidative stress, undermining immune function (Ni et al., 2022; Soares and Hamza, 2016).

Ferritin heavy chain 1 (FTH1), a key iron storage protein, helps mitigate iron toxicity by forming a ferritin complex with the light chain (FTL), which sequesters excess iron (Torti and Torti, 2002). FTH1 plays a complex role in the host’s immune response to *Mtb*. FTH1–deficient bone marrow–derived macrophages (BMDMs) or myeloid cells increase susceptibility to *Mtb* (Reddy et al., 2018; Khan et al., 2020), suggesting its protective role in host–anti-TB immune response. However, previous research has shown that the upregulation of FTH1 levels in macrophages is associated with TB disease progression (Dai et al., 2023). This paradox arises from *Mtb*’s ability to exploit ferritinophagy—a selective autophagic process regulated by NCOA4—to degrade FTH1, enhancing its intracellular survival (Dai et al., 2024; Mancias et al., 2014). Taken these together, maintaining a balanced FTH1 expression is essential for supporting immune function and preventing iron overload.

Ferritin expression, particularly FTH1, is tightly regulated at both transcriptional and translational levels (Torti and Torti, 2002). Cytokines such as TNF-α and IL-1 transcriptionally upregulate *FTH1* in response to stress and inflammation (Pham et al., 2004; Torti et al., 1988; Wei et al., 1990). Iron regulatory proteins (IRPs) bind to the iron–responsive element (IRE) in the 5′ untranslated region (5′UTR) of *FTH1* mRNA, controlling its translation (Hentze et al., 1987; Leibold and Munro, 1988). However, whether there are other molecular mechanisms governing *FTH1* expression, particularly in the context of *Mtb* infection, remain poorly understood.

Given that previous studies have identified two distinct *FTH1* transcripts with different 3′UTRs (Joshi et al., 1995), we suggest that alternative polyadenylation (APA) regulation may play a role in *Mtb*-driven *FTH1* expression post-transcriptionally. APA regulation generates mRNA isoforms with distinct 3′UTRs, which affect mRNA stability, translation, and localization (Mitschka and Mayr, 2022). APA is regulated by cleavage and polyadenylation (CPA) machinery and related cis-elements, including the polyadenylation signal (PAS) and downstream (G+U)-rich or U-rich region, which are recognized by factors like CPA specificity factor (CPSF) and cleavage stimulation factor (CSTF). These factors recruit proteins, including cleavage factor I (CFI), CFII and Poly(A) polymerase (PAP), to define functional PASs and facilitate RNA cleavage and polyadenylation. The UGUA motif—a highly prevalent cis-element upstream of polyadenylation site of human and mouse genes—is recognized by NUDT21 (also known as CPSF5, CFIm 25) (Yang et al., 2010). NUDT21, together with CPSF6 (also known as CFIm 68), forms CFI, which regulates the selection between proximal ((pA)p) and distal ((pA)d) polyadenylation sites (Yang et al., 2011). This regulation leads to 3′UTRs of varying lengths, which may contain regulatory elements such as microRNA or RNA-binding protein (RBP) binding sites. Dysregulated poly(A) site usage has been linked to many immune–related diseases—3′UTR shortening is predominantly exhibiting in solid tumors—suggesting that APA may contribute to disease etiology (An et al., 2021; Gruber and Zavolan, 2019; Li et al., 2023).

In this study, we reveal the role of APA in *Mtb*-driven *FTH1* expression in macrophages. Our findings demonstrate that *Mtb* infection reprograms *FTH1* APA, promoting the production of longer isoforms that enhance protein translation. We also highlight the involvement of NUDT21 in this process, underscoring the importance of NUDT21-mediated APA regulation in macrophage responses to *Mtb*. These insights deepen our understanding of host–pathogen interactions in macrophages during *Mtb* infection and highlight the need for further exploration of the APA regulation pathway in TB disease, particularly in relation to its regulators and their impact on disease progression.

## Materials and methods

### Bacteria strains and cell lines

The mycobacterial strains H37Ra (ATCC 25177), its fluorescent engineered version GFP-H37Ra as described previously (Mo et al., 2022), and Bacillus Calmette-Guérin (BCG Danish Strain 1331 sub-strain, NIBSC, SRNC-07/270) were cultured in Middlebrook 7H9 broth (BBL Microbiology Systems) supplemented with 10% oleic acid–albumin–dextrose–catalase (OADC; BD BBL), 0.05% (v/v) Tween-80 (Sigma), and 0.2% (v/v) glycerol (Sigma) or on Middlebrook 7H10 plates (BD Difco) containing 0.5% (v/v) glycerol and 10% OADC. Bone marrow-derived macrophages (BMDMs) were isolated from C57BL/6 mice (The Jackson Laboratory, Stock#000664), as described previously (Dai et al., 2023), and cultured for 1 week in DMEM supplemented with 20% L929-conditioned medium, 1 mM sodium pyruvate, 2 mM L-glutamine, and 10% FBS. Human monocytic THP-1 cells (TIB-202, ATCC) (4x10^5^ cells/mL) were seeded in 6- or 12-well plates (Costar) and differentiated with 20 ng/ml PMA (Sigma-Aldrich) for 24 h. Differentiated THP-1 macrophages and BMDMs were maintained in fresh pre-warmed media until further use. HEK293T and A549 cell lines were obtained from the Cell Bank of the Chinese Academy of Sciences (Shanghai, China), while RAW264.7 cells were a gift from the Center Lab of Longhua Branch, Shenzhen People’s Hospital. These cells were cultured in DMEM (Corning, USA) supplemented with 10% FBS (Gibco, Life Technologies) at 37°C in 5% CO_2_ until further use.

### *Mtb* infection

Bacteria harvested in the mid-log phase were washed and resuspended in PBS. The multiplicity of infection (MOI) was calculated based on bacterial optical density (OD) and confirmed by plating serial dilutions on Middlebrook 7H10 agar plates to determine the colony-forming units (CFU). PMA-differentiated THP-1 macrophages were infected with *Mtb* H37Ra or GFP-H37Ra at an MOI of 10 for 6 h, followed by three PBS washes to remove extracellular bacteria. Fresh medium was added, and cells were incubated for the indicated time points. For intracellular survival analysis, CFU assays were performed as described previously (Mo et al., 2022).

### Construction of fluorescent reporter plasmids

The 3′UTRs of *FTH1* transcripts were cloned into a pEGFP-C1 vector (Addgene, Plasmid #36412) downstream of the GFP ORF to create 3′UTR-Long (L) and 3′UTR-Short (S) plasmids. Scrambled sequences replaced the first or last 50 nucleotides of the upstream sequence element (USE) preceding the proximal (USE1) or distal (USE2) polyadenylation sites, generating pap-USE-F50, pap-USE-R50, pad-USE-F50 and pad-USE-R50 plasmids. A 25-nucleotide region of the downstream sequence element (DSE) was replaced with a scrambled sequence to create pap-DSE-F25 and pap-DSE-R25 plasmids. UGUA motif mutations were introduced by replacing TGTA with altered nucleotides (TAAA for UGUA motif 1; GGCG for UGUA motif 2) based on plasmid 3′UTR-L.

### 3′RACE

Total RNA was extracted from *Mtb*-infected or uninfected macrophages and fluorescent plasmids transfected eukaryotic cells, using RNeasy kits (Omega). First-strand cDNA was synthesized from 1 µg of RNA using the following 3′ap primer:

5′-GCTGTCAACGATACGCTACGTAACGGCATGACAGTGtttttttttttttttttt-3′

The 20 µL reaction mix contained 1 mM dNTPs, 1X RT buffer, 0.1 M DTT, and 200 U of MMLV Reverse Transcriptase, and was incubated at 42°C for 60 min before enzyme inactivation at 70°C for 5 min.

For the first PCR amplification, 2 µL of cDNA was amplified using 0.2 µM of each of the following primers:

*FTH1* forward: 5′-ATGACCCCCATTTGTGTGACTTCATTGAG-3′;

*Fth1* forward: 5′-TACGTCTATCTGTCTATGTCTTGTT-3′;

3′ap-CA reverse: 5′-GCTGTCAACGATACGCTACGTAACGGCATGACAGTGttttttttttttttttttCA-3′.

The reaction was completed in 1X PCR buffer, with 0.2 mM dNTPs and 2.5 U Taq polymerase. The PCR conditions were as follows: 95°C for 3–5 min, followed by 35 cycles of 95°C for 30 sec, 55°C for 30 sec, and 72°C for 1 min/kb, with a final extension at 72°C for 10 min. A second PCR was performed using 1 µL of the first PCR product and the following primers:

*FTH1* forward: 5′-GCCTCGGGCTAATTTCCC-3′;

*Fth1* forward: 5′-CTCATGAGGAGAGGGAGCAT-3′;

3′NestedPrimer reverse: 5′-CGCTACGTAACGGCATGACAGTG-3′.

Products were separated by agarose gel electrophoresis and then gel-purified and sequenced.

### siRNAs and cell transfection

For siRNA transfection, siFTH1 (targeting all *FTH1* transcripts; RiboBio, siG2010191151216466), siFTH1-L (targeting the longer *FTH1* isoform; RiboBio, siG2010191151217558), and siNUDT21 (targeting the human *NUDT21* gene; RiboBio, stB0001835B) were transfected into THP-1 macrophages, while siNudt21 (targeting the mouse *Nudt21* gene; RiboBio, siG170614045522) was transfected into BMDMs, using Lipofectamine RNAiMAX (Invitrogen) following the manufacturer’s protocol. Scrambled siRNAs (siNC) were used as negative controls. For plasmid transfection, cells at 60–80% confluency were transfected using Lipofectamine 3000 (Invitrogen) in serum-free medium, and according to the manufacturer’s instructions. Cells were harvested 24–48 h later for further analyses. Knockdown efficiency was assessed by western blotting 36–48 h post-transfection.

### qPCR

Total RNA was extracted using RNeasy kits (Omega) and cDNA was synthesized from 1 µg of total RNA using olig(dT) primer. A total of 1 μl cDNA was then analyzed by quantitative PCR on a 7500 Fast Real-Time PCR System (Thermo Fisher Scientific) using SYBR Green PCR Master Mix (TaKaRa). The following primers were used:

*FTH1* forward: 5′-AGAACTACCACCAGGACTCA-3′*;*
*FTH1* reverse: 5′-TCATCGCGGTCAAAGTAGTAAG-3′;
*FTH1-L* forward: 5′-GCCGTTGTTCAGTTCTAATCACA-3′;
*FTH1-L* reverse: 5′-CCAAGACAGCCACACCTTAGT-3′;
*GFP* forward: 5′-AGGACGACGGCAACTACAAG-3′;
*GFP* reverse: 5′-TTGTACTCCAGCTTGTGCCC-3′;
*GAPDH* forward: 5′-ACCACAGTCCATGCCATCAC-3′;
*GAPDH* reverse: 5′-TCCACCACCCTGTTGCTGTA-3′).

Statistical analyses were performed using GraphPad Prism version 8 (GraphPad Software Inc.). Statistical significance between groups was assessed using one-way ANOVA with Tukey’s *post hoc* test (for two data groups) or two-way ANOVA with Bonferroni’s *post hoc* test (for three data groups). A p-value < 0.05 was considered statistically significant. Detailed statistical information is provided in the figure legends.

### Western blotting

Cells were lysed in ice-cold RIPA buffer (Thermo Fisher Scientific) supplemented with protease inhibitors (Roche). Protein concentrations were determined using a BCA Protein Assay Kit (Thermo Fisher Scientific). Equal amounts of protein (30 µg) were separated by SDS-PAGE, transferred onto PVDF membranes (Merck Millipore), and blocked with 5% non-fat milk in PBST (PBS with 0.1% Tween-20) for 1 hour. Membranes were incubated overnight at 4°C with primary antibodies against FTH1 (Abcam, ab75973; Santa Cruz Biotechnology, sc-376594), NUDT21 (Proteintech, 66335-1-Ig), CPSF6 (Abcam, ab99347), GFP-tag (Proteintech, 50430-2-AP), H3 (Abcam, ab176842), and β-actin (Abcam, ab179467), according to the manufacturer’s instructions. Afterward, membranes were incubated for 1 hour at room temperature with HRP-conjugated anti-rabbit or anti-mouse IgG secondary antibodies (Abcam, ab205718, ab205719). After washing, protein bands were detected using ECL reagent (Thermo Fisher Scientific) following the manufacturer’s protocol and visualized using a Chemiluminescence Image System (Minichem™, China), as previously described (Mo et al., 2022).

For quantitation, protein band intensities were measured using ImageJ software. The integrated density of each target protein band was normalized to its corresponding loading control (e.g., H3 or β-actin) to account for variations in protein loading. The relative band intensities shown below each image were quantified from the specific experiment presented in the figure. Additionally, multiple independent experiments were performed to confirm reproducibility.

### *In vitro* transcription and RNA pull-down

A tRNA scaffolded Streptavidin Aptamer (tRSA) was incorporated into full-length (tRSA-3′UTR-L) and shorter (tRSA-3′UTR-S) 3′UTRs of *FTH1* mRNA and prepared using T7 RNA polymerase as described previously (Iioka and Macara, 2015). RNA was purified using TRIzol reagent (Life technologies, cat#15596-026) according to the manufacturer’s protocol. For cell lysate preparation, *Mtb*-infected or uninfected THP-1–differentiated macrophages were harvested and lysed in lysis buffer (10 mM HEPES pH 7.0, 200 mM NaCl, 10 mM MgCl_2_, 1% TritonX-100, 1 mM DTT) supplemented with protease inhibitors and an RNase inhibitor (Promega, cat#N2115). The lysate was centrifuged, and the supernatant was collected. Protein concentrations in the lysate were determined using the BCA Protein Assay Kit (Thermo Fisher). The tRSA–tagged RNA (10 µg) was incubated with 200 µg of cell lysate for 2 hours at 4°C with gentle rotation to promote RNA-protein binding. Streptavidin-coated magnetic beads (Invitrogen, 65605D) were then added to capture RNA–protein complexes. After a 1-hour incubation, the beads were washed five times with cold lysis buffer to remove nonspecific binding. The bound RNA–protein complexes were eluted by boiling the beads in 2X SDS-PAGE sample buffer and applied for western blot analysis.

### Immunoprecipitation

Cells were lysed in lysis buffer (Beyotime, P0013) containing a protease inhibitor cocktail (Roche). For immunoprecipitation, 1 mg of total protein was incubated overnight at 4°C with 2 µg of anti-NUDT21, anti-CPSF6 or control IgG. The mixture was then incubated for additional 2 hours with 50 µl of protein A/G magnetic beads (Santa Cruz Biotechnology) at 4°C with continuous rotation. The beads were washed, and bound proteins were eluted by boiling the samples in SDS-PAGE sample buffer prior to western blotting analysis.

### Immunostaining and confocal microscopy

THP-1 macrophages (2 × 10^5^ cells/ml) infected with GFP-H37Ra (MOI = 10) were fixed with 4% (v/v) formaldehyde for 15 min and permeabilized with 0.2% (v/v) Triton X-100 for 5 min at room temperature. The cells were then blocked with 5% BSA and incubated in the dark with NUDT21 or CPSF6 primary antibodies overnight. After washing, the samples were incubated in the dark for 1 hour with Alexa Fluor-conjugated secondary antibodies (Invitrogen, anti-rabbit Alexa fluor 555, 1:200), and Hoechst (Invitrogen; 10 ng/ml) for 5 min at room temperature before visualization. Images were captured under an Olympus FV1000 confocal microscope (NIKON A1R).

### Nuclear isolation

Macrophages were lysed in ice-cold Nuclei Isolation Buffer (20 mM Tris-HCl, pH 7.4, 150 mM NaCl, 1 mM EDTA) with protease inhibitors. After mechanical homogenization, the nuclei were pelleted by centrifugation at 1,000 g for 10 min at 4°C. The supernatant and resuspended nuclei were isolated, boiled in SDS sample buffer, and analyzed by western blotting.

### CFU assay

BMDMs were infected with *Mtb* at an MOI of 10 and incubated for 6 h at 37°C to allow for bacterial uptake. After infection, the macrophages were washed three times with PBS to remove non-internalized bacteria and further incubated at 37 °C in 5% CO_2_ for 72 h. To quantify intracellular bacteria, macrophages were lysed with 0.1% Triton X-100 (Sigma-Aldrich) in PBS for 15 min at room temperature. The lysates were then serially diluted in PBS and plated in triplicate on Middlebrook 7H10 agar plates. Plates were incubated at 37°C for 3–4 weeks to allow for colony formation. CFU counts were performed at 6-h and 72-h post-infection as described previously (Mo et al., 2022). Statistical analyses were performed using GraphPad Prism version 8 (GraphPad Software Inc.), with statistical significance of differences between groups was determined using a student’s unpaired t test.

### Study approval

The present study was approved by the Ethics Committees of Wuhan Polytechnic University (Wuhan, China), Shenzhen University (Shenzhen, China), and Shenzhen People’s Hospital (Shenzhen, China). The study was conducted using BMDMs isolated from animals and did not involve *in vivo* experiments. However, we acknowledge the ARRIVE guidelines and adhere to ethical principles for animal research. All procedures were performed in compliance with the ethical guidelines set by the Wuhan Polytechnic University Animal Ethics Committee, following relevant institutional and national regulations for the care and use of laboratory animals.

## Results

### *Mtb* infection alters the *FTH1* APA profile in macrophages

**Figure 1:**
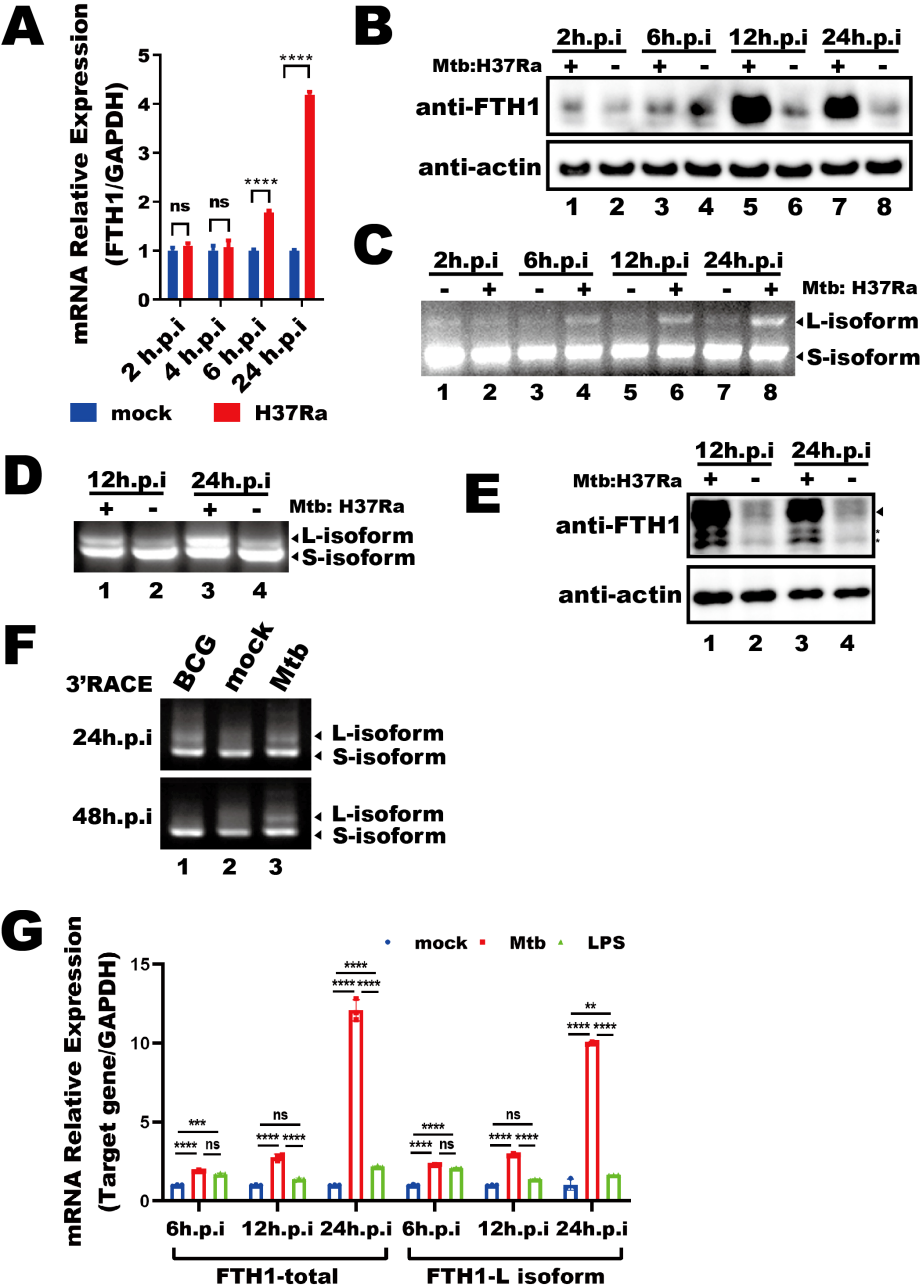
*Mtb* infection alters the *FTH1* APA profile in macrophages. **(A)** qPCR analysis of *FTH1* mRNA in *Mtb*-infected (H37Ra) or uninfected (mock) macrophages for 2-, 4-, 6-, and 24-hours post-infection. Data are normalized to GAPDH and presented as fold change relative to the control. Error bars represent the mean ± SEM from two independent experiments. Statistical significance was determined by two-way ANOVA with Sidak’s multiple comparisons test; ****P < 0.0001. **(B)** Western blot analysis of FTH1 protein expression in THP-1 macrophages infected with *Mtb* at 2, 6, 12, and 24 hours. β-actin was used as a loading control. **(C)** Agarose gel electrophoresis of 3′RACE products from *Mtb*-infected or uninfected (control) macrophages. Bands representing different *FTH1* transcript isoforms are indicated by arrows. **(D)** 3′RACE assay of *Fth1* transcripts and **(E)** western blot analysis of mouse FTH1 protein expression in *Mtb*-infected or uninfected RAW264.7 cells. The specific FTH1 band is marked by an arrow, and nonspecific bands are indicated with stars. β-actin was used as a loading control. **(F)** Agarose gel electrophoresis of *FTH1* 3′RACE products from macrophages infected with BCG or *Mtb* (H37Ra) at 24- and 48-hours post-infection. **(G)** qPCR analysis of total and longer *FTH1* transcripts in THP-1 macrophages infected with *Mtb* (H37Ra, MOI = 10) or stimulated with LPS (20 ng/mL). GAPDH was used as the reference gene. Error bars represent the mean ± SEM from three independent experiments. Statistical significance was determined by two-way ANOVA with Tukey’s multiple comparisons test; **P < 0.01, ***P < 0.001 ****P < 0.0001. Three independent experiments were performed to ensure reproducibility, and representative images were selected for presentation. ns, not significant.

We first investigated how *Mtb* infection affects *FTH1* expression in THP-1 macrophages. These cells were infected with the H37Ra *Mtb* strain or left uninfected, and total RNA were collected at 2, 4, 6 and 24 hours post infection. We observed an increase in *FTH1* mRNA levels over time in the infected cells compared with the uninfected cells (**Figure 1A**), with a corresponding rise in protein abundance (**Figure 1B**). To explore whether APA contributes to this upregulation, we analyzed the APA profile of *FTH1* in *Mtb*-infected and uninfected THP-1 macrophages using a 3′RACE assay. Interestingly, *Mtb* infection shifted the APA profile toward the longer *FTH1* transcript isoforms (**Figure 1C**), indicating the potential involvement of APA in this *Mtb*-driven upregulation of FTH1 expression. To determine if this mechanism is conserved across species, we performed the same 3′RACE assay on RAW264.7 murine macrophages and observed a similar shift toward longer *Fth1* isoforms and enhanced protein expression (**Figures 1D, E**). These findings indicate that *Mtb* infection triggers *FTH1* APA regulation in both human and murine macrophages.

To evaluate the specificity of the *Mtb*-driven shift in *FTH1* APA, we stimulated THP-1 macrophages with lipopolysaccharide (LPS) or Bacillus Calmette-Guérin (BCG). While BCG stimulation initially increased the expression of longer *FTH1* transcript isoforms at 24 hours, this increase diminished by 48 hours post-infection, returning to baseline levels compared with uninfected THP-1 macrophage (**Figure 1F**, lane 1 vs. lane 2). In contrast, *Mtb* infection induced a sustained, robust increase in longer *FTH1* transcript isoforms at 48 hours in THP-1 macrophages (**Figure 1F**, lane 3). Additionally, LPS treatment led to only a modest increase in longer *FTH1* transcript isoforms (FTH1-L) at 24 hours, much less than the response seen in *Mtb*-infected THP-1 macrophages (**Figure 1G**). Together, these results demonstrate that *Mtb* infection specifically induces a unique *FTH1* APA regulation, resulting in enhanced expression of FTH1-L in macrophages.

### *Mtb*-induced longer *FTH1* transcripts enhance protein expression

**Figure 2:**
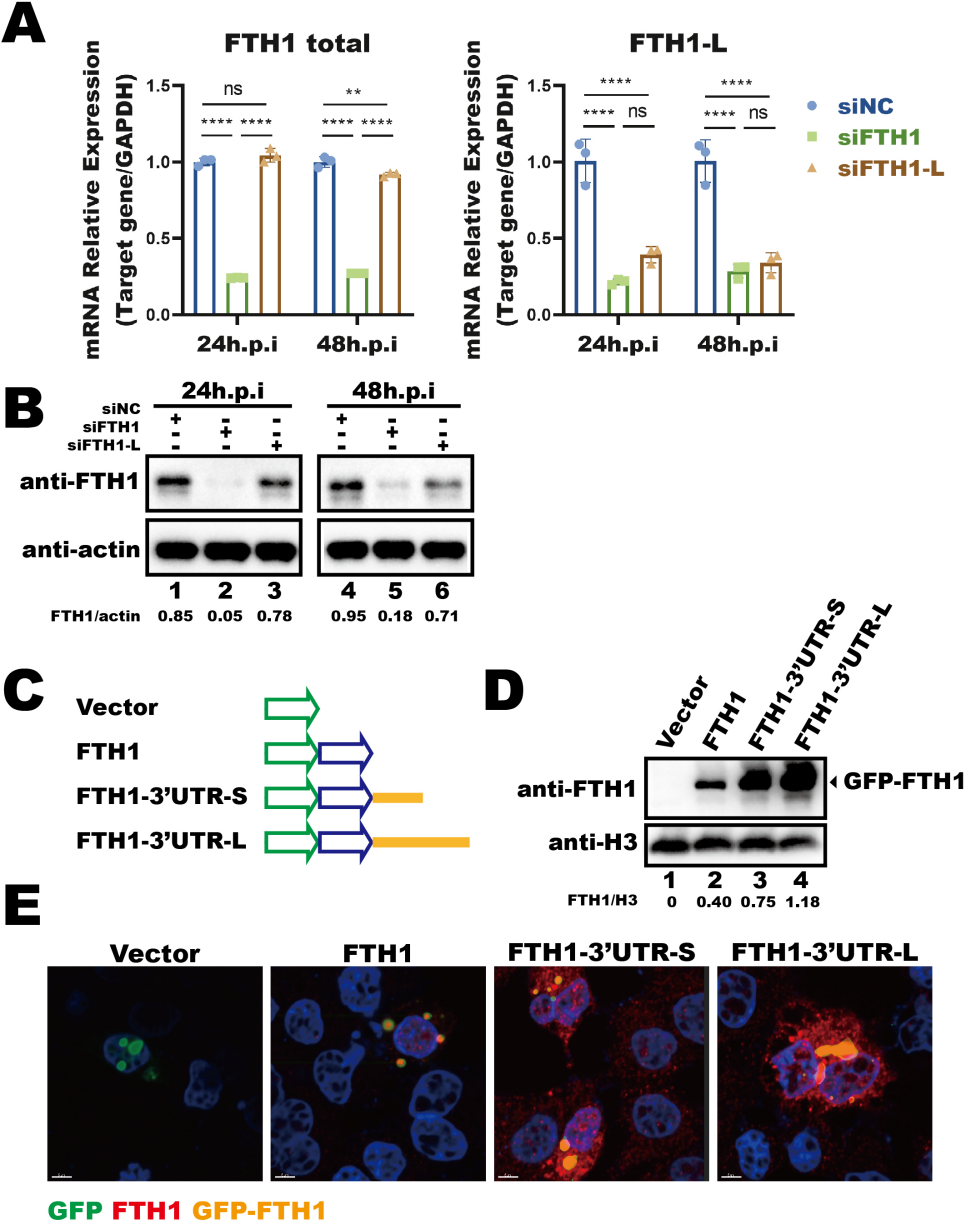
*Mtb*-induced longer *FTH1* transcripts enhance protein expression. **(A)** qPCR analysis of siRNA knockdown efficiency (siFTH1, green; siFTH1-L, brown) in THP-1 macrophages harvested 24- or 48-hours post-transfection. Total RNA (1 µg) was used for cDNA synthesis, followed by qPCR to measure total *FTH1* (left panel) or the longer isoform–specific transcripts (right panel). Data are normalized to GAPDH and presented as fold change relative to the control. Error bars represent the mean ± SEM from three independent experiments. Statistical significance was determined by two-way ANOVA with Tukey’s multiple comparisons test; **P < 0.01, ****P < 0.0001. **(B)** Western blot analysis of FTH1 expression in THP-1 macrophages transfected with siRNAs targeting total *FTH1* mRNA (siFTH1) or the longer isoform (siFTH1-L). β-actin served as a loading control. The relative abundance of FTH1 (normalized to actin) is shown below each lane. The quantification represents the result of a single experiment. **(C)** Schematic of the GFP-tagged FTH1 expression constructs used to evaluate the 3′UTR’s role in *FTH1* expression. **(D)** Western blot analysis was performed to assess GFP-FTH1 protein expression in A549 cells transfected with 3′UTR-FTH1 overexpression plasmids shown in **Figure 3C**. Protein levels were quantified using ImageJ, with target protein band intensity normalized to the corresponding loading control. The relative abundance of FTH1 (normalized to H3) is shown below each lane. The quantification represents the result of a single experiment. **(E)** Confocal microscopy of GFP-FTH1 in A549 cells 24 hours post-transfection to assess its subcellular distribution. Three independent experiments were performed to ensure reproducibility, and representative images were selected for presentation. ns, not significant.

We next investigated the impact of this longer *FTH1* transcript on protein expression. The siRNAs targeting the longer *FTH1* isoform (FTH1-L) or total *FTH1* transcripts were transfected into THP-1 macrophages 24 hours prior to *Mtb* infection, and *FTH1* mRNA levels (**Figure 2A**) and protein abundance were assessed at 24- and 48-hours post-infection (**Figure 2B**). Silencing FTH1-L strongly reduced FTH1 protein levels in *Mtb*-infected THP-1 macrophages at 48 hours (**Figure 2B**, lane 6 vs. lane 4), highlighting the role of FTH1-L in the upregulation of FTH1 expression in these cells.

To further investigate the impact of the longer transcript isoform, induced by *Mtb*-driven APA regulation, on protein translation, we examined the role of 3′UTR in modulating FTH1 translation. We constructed FTH1 overexpression plasmids based on a fluorescent vector, in which the FTH1 coding sequence was fused with either the longer or shorter *FTH1* 3′UTR isoform (**Figure 2C**). Upon transfection into A549 cells, both 3′UTRs enhanced translation of the GFP–FTH1 fusion protein compared to the FTH1 plasmid without a 3′UTR (**Figure 2D**, lane 3 and lane 4 vs. lane 2). Notably, the longer *FTH1* 3′UTR clearly enhanced protein translation efficiency compared to the shorter isoform, resulting in increased GFP–FTH1 protein abundance (**Figure 2D**, lane 4 vs. lane 3). These results suggest that the 3′UTR length is linked to the translational efficiency of *FTH1* transcripts. However, 3′UTR length did not affect the distribution of FTH1 protein in transfected cells, which was predominantly localized in the cytoplasm (**Figure 2E**, overexpressed GFP–FTH1 highlighted in orange). Overall, our findings indicate that the *Mtb*-induced shift in *FTH1* APA increases the abundance of FTH1-L isoform, leading to enhanced FTH1 protein production.

### The longer *FTH1* isoform contains essential APA regulatory *cis*-elements

**Figure 3:**
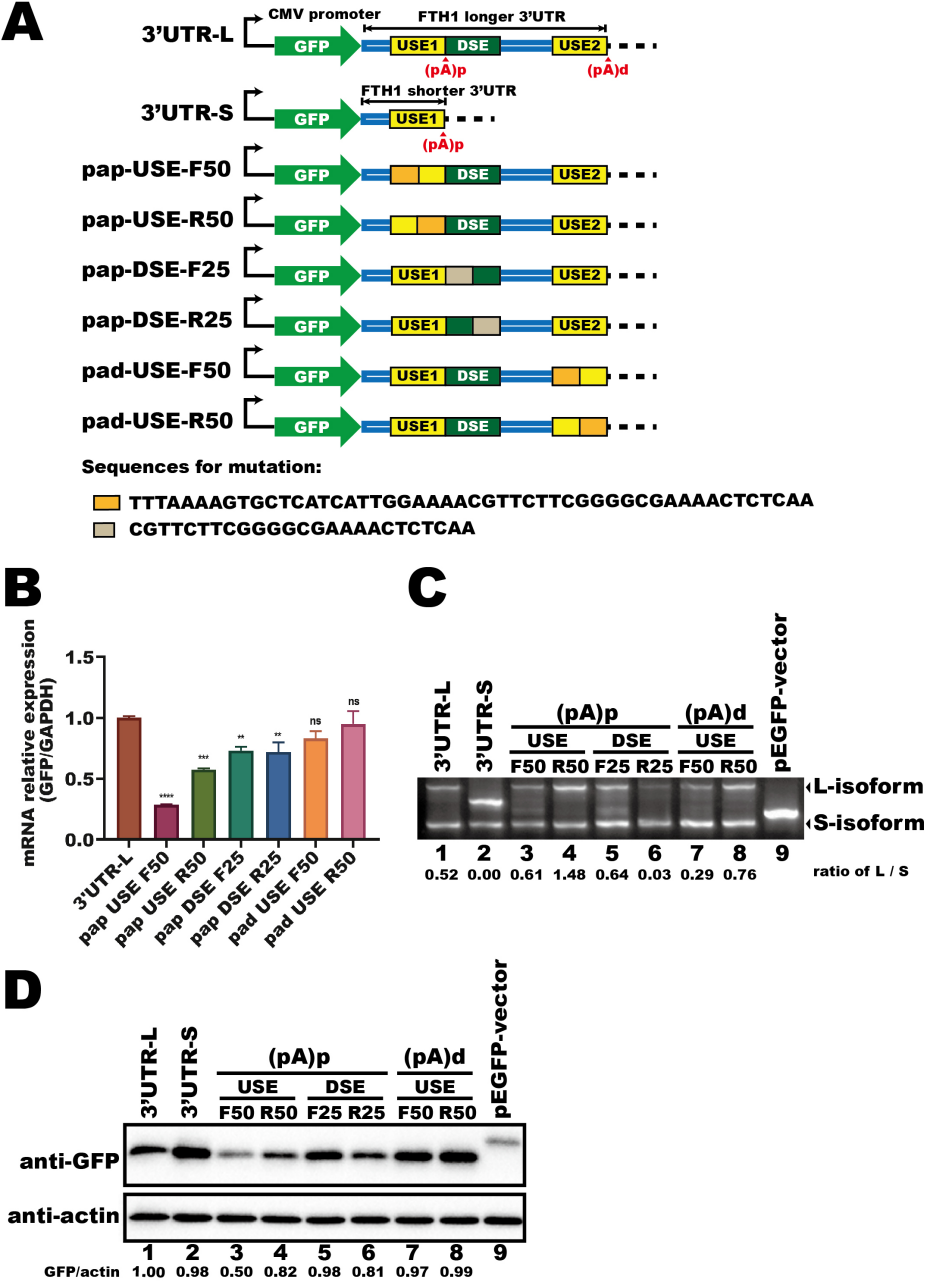
The longer *FTH1* isoform contains essential APA regulatory cis-elements. **(A)** Schematic of the fluorescent reporter plasmids (GFP) containing *FTH1* 3′UTRs and mutant constructs. Sequences used for replacement are indicated beneath the schematic. **(B)** qPCR analysis of fluorescent gene expression in HEK293T cells transfected with plasmids as shown in **Figure 3A**. Data are normalized to GAPDH and presented as fold change relative to the control. Error bars represent the mean ± SEM from three independent experiments. Statistical significance was determined by one-way ANOVA with Dunnett’s multiple comparisons test; **P < 0.01, ***P < 0.001, ****P < 0.0001. **(C)** 3′RACE assay of fluorescent transcripts in HEK293T cells transfected with plasmids shown in **(A)**. RNA abundances were quantified using ImageJ software, and the numerical values below the blot represent the ratio of the longer isoform (L-isoform) to the shorter isoform (S-isoform). **(D)** Western blot analysis of GFP protein levels in HEK293T cells transfected with plasmids shown in **(A)**. Protein levels were quantified using ImageJ software. The intensity of the target band was normalized to the corresponding loading control. Numerical values representing the target protein (GFP) abundance relative to the loading control (β-actin) are shown at the bottom of each lane. Three independent experiments were performed to ensure reproducibility, and representative images were selected for presentation. The difference in molecular weight arises from the insertion of the 3′UTR, which introduced a stop codon, producing a shorter ~26 kDa fluorescent protein, whereas the native vector expresses a ~29 kDa protein. ns, not significant.

To determine the *cis*-acting elements within the 3′UTR that regulate FTH1-L isoform expression, we examined how specific cis-elements within the 3′UTR influence APA regulation. We cloned either the longer (3′UTR-L) or shorter (3′UTR-S) *FTH1* 3′UTR into a fluorescent reporter vector (**Figure 3A**). The upstream element (USE1) of the (pA)p site, which is common to both isoforms, was replaced with an unrelated sequence of the same length. In addition, the upstream element (USE2) of the (pA)d site and the downstream element (DSE), both exclusive to the 3′UTR-L, were substituted with unrelated sequences (**Figure 3A**).

Following HEK293T cell transfection, we measured mRNA expression, protein levels, and the APA profile of the fluorescent gene. Mutations near the (pA)d site (pad-USE-F50 and pad-USE-R50) did not markedly alter mRNA levels compared to 3′UTR-L (**Figure 3B**). However, pad-USE-R50 generated a higher proportion of L-isoform transcripts than pad-USE-F50 (**Figure 3C**, lane 8 vs. lane 7), leading to a modest increase in GFP protein levels (**Figure 3D**, lane 8 vs. lane 7). In contrast, DSE mutations caused a ~15% reduction in fluorescent mRNA expression (**Figure 3B**, pap-DSE-F25 and pap-DSE-R25 vs. 3′UTR-L). Among these, pap-DSE-F25, in which the first 25 nucleotides were replaced with a scrambled sequence, increased L-isoform abundance (**Figure 3C**, lane 5 vs. lane 6) and enhanced GFP protein expression (**Figure 3D**, lane 5 vs. lane 6) compared to pap-DSE-R25. Notably, pap-DSE-R25 exhibited the strongest reduction in L-isoform expression (**Figure 3C**, lane 6 vs. lane 1), while pap-DSE-F25 showed a slight increase (**Figure 3C**, lane 5 vs. lane 1). These differences further support the idea that the longer isoform has higher translation efficiency.

Mutations in USE1, near the (pA)p site, caused a more pronounced reduction in mRNA expression, particularly in pap-USE-F50 (**Figure 3B**, pap-USE-F50 and pap-USE-R50 vs. 3′UTR-L). Moreover, mutating the last 50 nucleotides of USE1 (pap-USE-R50) doubled L-isoform abundance (**Figure 3C**, lane 4 vs. lane 3) and elevated GFP protein levels (**Figure 3D**, lane 4 vs. lane 3) compared to pap-USE-F50. These findings highlight the importance of cis-elements near the (pA)p site, especially within the final 50 nucleotides of USE1, in regulating *FTH1* expression. We propose that trans-acting factors binding to USE1 might mediate *Mtb*-induced APA regulation of *FTH1*.

### *Mtb* infection reduces the interaction between NUDT21 and *FTH1* transcripts in THP-1 macrophages

**Figure 4:**
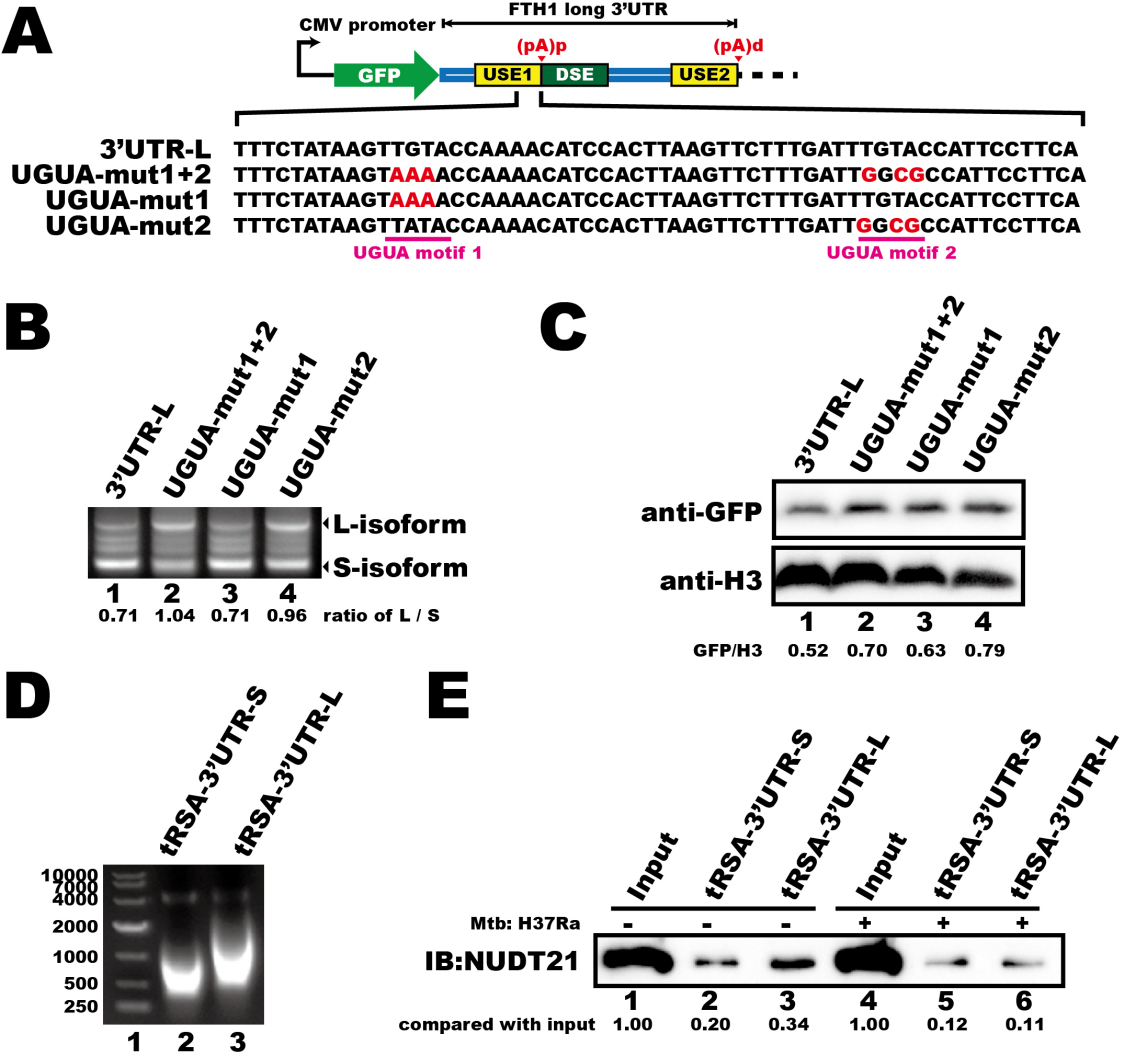
*Mtb* infection reduces the interaction between NUDT21 and *FTH1* transcripts in THP-1 macrophages. **(A)** Schematic of the fluorescent reporter plasmids (GFP) containing *FTH1* 3′UTR and UGUA motif–mutated constructs. Mutated nucleotides are highlighted in red. **(B)** 3′RACE of GFP transcripts and **(C)** Western blot analysis of GFP protein levels demonstrate how UGUA motif mutations affect the APA profile and protein expression of the fluorescent gene. RNA and protein abundance was quantified using ImageJ software, with normalized numerical values displayed at the bottom of the respective panels. **(D)** Agarose gel electrophoresis of *in vitro*–transcribed, tRSA-tagged *FTH1* 3′UTR constructs representing the short (lane 2) and the long (lane 3) versions. **(E)** RNA pull-down demonstrating the interaction between NUDT21 and the *FTH1* 3′UTRs in *Mtb*-infected or uninfected THP-1 macrophages. Protein abundance was quantified using ImageJ software, and the raw values are shown at the bottom. Three independent experiments were performed to ensure reproducibility, and representative images were selected for presentation.

Having established that the last 50 nucleotides of the USE1 in *FTH1* 3′UTR contains key cis-elements involved in regulating gene expression, we next further investigated what is it and how it participates the APA regulation of FTH1 during *Mtb* infection. We identified two UGUA motifs within this region and assessed their functional significance by creating mutant plasmids based on the 3′UTR-L fluorescent vector (**Figure 4A**). We found that mutating both motifs altered polyadenylation site selection, resulting in an increased abundance of the longer isoform (**Figure 4B**, lane 2 vs. lane 1). Mutating the UGUA motif closest to the (pA)p site (UGUA–mut2) also increased longer isoform mRNA (**Figure 4B**, lane 4 vs. lane 1), while mutation of the other UGUA motif (UGUA–mut1) had no significant effect on the proportion of longer isoform mRNA (**Figure 4B**, lane 3 vs. lane 1). Moreover, mutations in either UGUA motif or in both motifs resulted in a slight increase in protein expression compared to the wild-type (**Figure 4C**), suggesting that the UGUA motifs within the *FTH1* 3′UTR are critical for modulating APA and gene expression.

NUDT21 is a key regulator of the APA pathway that interacts with UGUA motifs (Yang et al., 2010). Therefore, we investigated whether *Mtb* infection interferes with this interaction. We performed RNA pull-down assays using *in vitro*–transcribed, tRSA–tagged FTH1–3′UTR RNAs (**Figure 4D**) and lysates from *Mtb*-infected or uninfected THP-1 macrophages. *Mtb* infection reduced NUDT21 binding to both FTH1–3′UTRs (**Figure 4E**, lane 2 vs. lane 5; lane 3 vs. lane 6), with a particularly pronounced decrease in affinity for the longer transcript isoform (**Figure 4E**, lane 6 vs. lane 3). We propose that this disruption likely contributes to the *Mtb*-induced *FTH1* APA shift and the subsequent upregulation of FTH1-L.

### *Mtb* infection disrupts the interactions between NUDT21 and CPSF6

**Figure 5:**
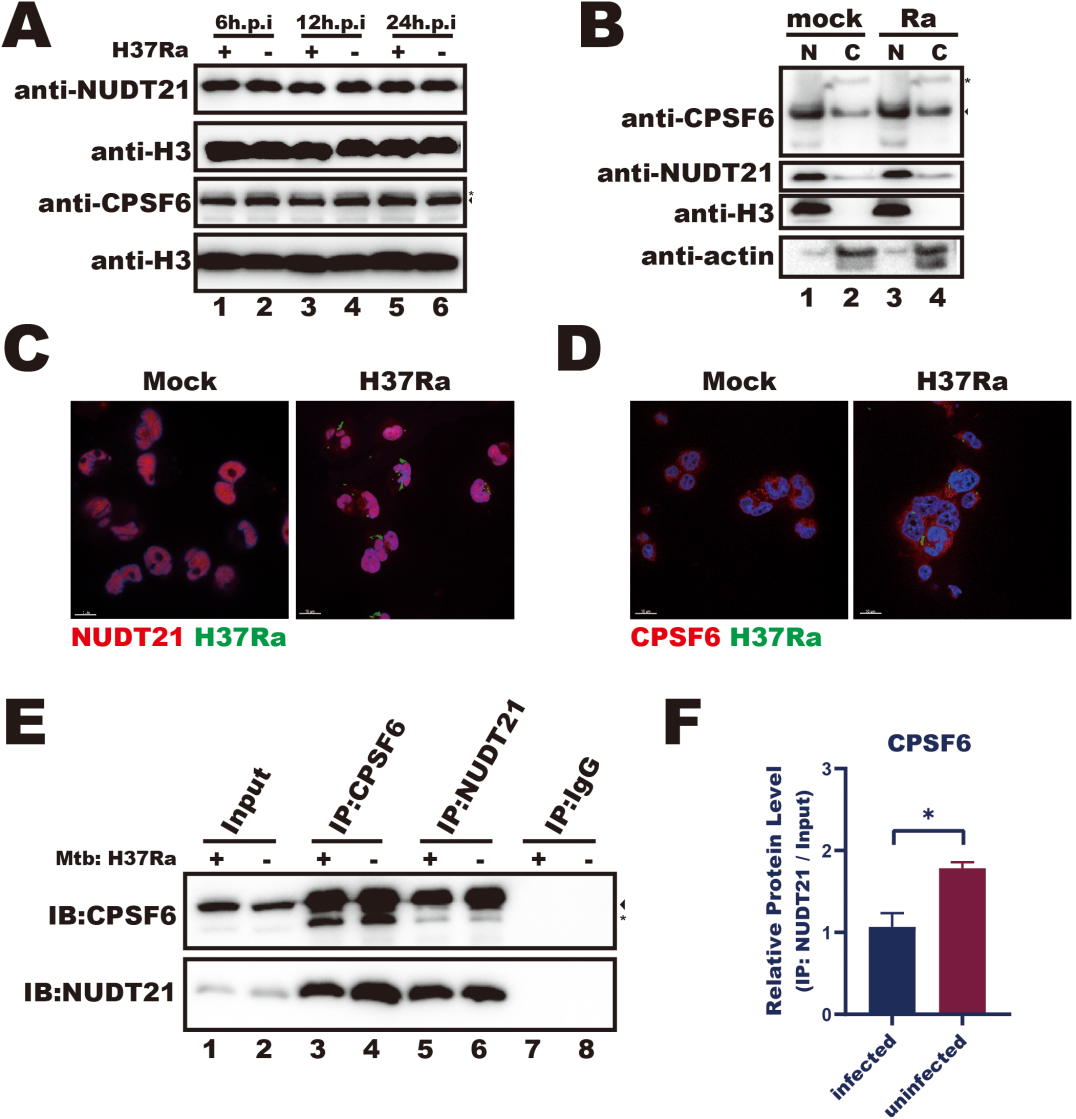
*Mtb* infection disrupts the interactions between NUDT21 and CPSF6. **(A)** Western blot analysis of NUDT21 and CPSF6 protein levels in THP-1 macrophages infected with *Mtb* for 6, 12, and 24 hours or left uninfected (control). H3 served as a loading control. **(B)** Western blot of nuclear (N) and cytoplasmic **(C)** fractions showing NUDT21 and CPSF6 distribution in *Mtb*-infected or uninfected THP-1 macrophages. H3 was used as a nuclear loading control, and β-actin was used as a cytoplasmic loading control. **(C)** Representative confocal microscopy images of THP-1 macrophages infected with *Mtb* (green) and immunolabeled for NUDT21 (red) or **(D)** CPSF6 (red). Hoechst dye was used to stain nuclei (blue). **(E)** Western blot analysis of immunoprecipitation (IP) products from *Mtb*-infected and uninfected THP-1 macrophages at 48 hours post-infection. IgG served as a negative control. The target protein band is marked by an arrow, and a nonspecific interaction band is indicated with a star. Representative images from three independent experiments are shown. **(F)** Quantification of protein band intensities from three independent IP experiments with THP-1 macrophages using ImageJ software. Statistical analysis was performed in GraphPad Prism 8, and differences between infected and uninfected groups were determined using unpaired t test. *P < 0.05.

Given that *Mtb* infection reduces the interaction between NUDT21 and the *FTH1* 3′UTR, we sought to investigate the underlying mechanisms. As the CFI complex, composed of NUDT21 and CPSF6, guides precise polyadenylation site selection (Yang et al., 2011), we next investigated whether *Mtb* infection affects the expression and cellular distribution of these regulatory proteins. During infection, NUDT21 protein levels remained unchanged (**Figure 5A**), and both western blotting and confocal microscopy showed no noticeable differences in its cellular distribution between infected and uninfected cells (**Figures 5B, C**). These results suggest that *Mtb*-driven *FTH1* APA regulation is more likely due to functional impairment of NUDT21 rather than alterations in its expression or localization.

We then focused on CPSF6, a key RNA-binding partner of NUDT21 within the CFI complex. Similar to NUDT21, *Mtb* infection did not affect CPSF6 expression or cellular distribution (**Figures 5A, B, D**). However, reciprocal co-immunoprecipitations assays revealed a significant reduction in the NUDT21–CPSF6 interaction in infected THP-1 macrophages (**Figures 5E, F**). This dissociation likely compromises the RNA recognition capability of the CFI complex, thereby contributing to APA dysregulation in *Mtb*-infected macrophages.

### siRNA-mediated NUDT21 knockdown impairs macrophage anti-*Mtb* function

**Figure 6:**
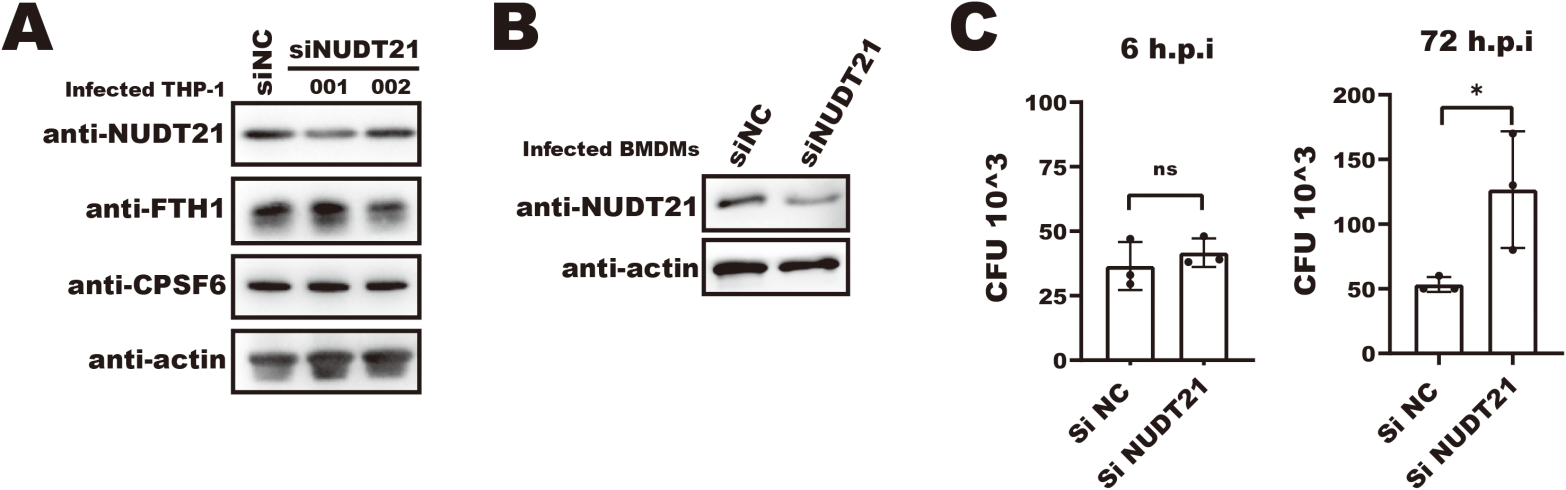
siRNA-mediated NUDT21 knockdown impairs macrophage anti-*Mtb* function. **(A)** Western blot analysis of FTH1 and NUDT21 expression in NUDT21 knockdown THP-1 macrophages. β-actin was used as a loading control. **(B)** Western blot showing siRNA-mediated NUDT21 knockdown in bone marrow–derived macrophages (BMDMs). β-actin was used as a loading control. **(C)** CFU assays of *Mtb* infected BMDMs treated with siNUDT21 or control siNC. Cells were infected with *Mtb* and harvested at 6- and 72-hours post-infection. After serial dilution and plating on 7H10 agar, the plates were incubated at 37°C, and colony-forming units (CFUs) were counted after 3 weeks. *P < 0.05.

Having shown that *Mtb* disrupts the interaction between key APA regulators, we next investigated the functional relevance of NUDT21 in macrophage defense against *Mtb*. To this end, we used siRNA to knock down NUDT21 expression in THP-1 macrophages, which led to a modest increase in FTH1 protein levels (**Figure 6A**, middle lane). However, the knockdown efficiency in this cell type was suboptimal, and no significant change in intracellular *Mtb* survival was observed (data not shown).

In contrast, siRNA-mediated NUDT21 knockdown in BMDMs resulted in a significantly higher intracellular *Mtb* burden at 72 hours post-infection (**Figures 6B, C**), underscoring the importance of NUDT21 in maintaining macrophage anti-*Mtb* function. Together, these findings suggest that *Mtb*-induced disruption of NUDT21 activity contributes to dysregulated APA of immune-relevant transcripts, which may in turn impair macrophage-mediated host defense.

## Discussion

FTH1 is crucial for maintaining iron homeostasis in macrophages and plays a vital role in the immune response to *Mtb* infection (Reddy et al., 2018). By sequestering excess intracellular iron, FTH1 limits its availability to *Mtb*, thereby hindering bacterial growth (Cassat and Skaar, 2013). However, *Mtb* can degrade FTH1 through the host ferritinophagy pathway, releasing iron to support its survival (Dai et al., 2024). These findings position FTH1 as a central mediator in balancing iron homeostasis within host–pathogen interactions during *Mtb* infection. In this study, we reveal a novel *Mtb*-driven NUDT21-mediated APA mechanism that enhances FTH1 expression in macrophages, adding a new layer of complexity to the regulation of iron homeostasis in these cells during *Mtb* infection.

The expression of *FTH1* is regulated through multiple pathways in response to infection. Notably, the transcription factor Nrf2 promotes iron storage by increasing ferritin levels (Kerins and Ooi, 2018), while pro-inflammatory cytokines such as IL-6, IL-1β, and TNF-α upregulate *FTH1* transcription (Rogers, 1996; Torti and Torti, 2002). However, the levels of these factors typically return to baseline due to host immune regulation, which aims to reduced excessive inflammation, or as a result of *Mtb*’s immune evasion mechanisms. Consequently, these pathways alone do not fully explain the sustained expression of FTH1 in *Mtb*-infected macrophages. At the translational level, the well-established IRP–IRE system regulates *FTH1* expression in response to intracellular iron levels (Wilkinson and Pantopoulos, 2014; Rouault, 2006). Yet, *Mtb*-infected macrophages exhibit significantly lower intracellular iron concentrations (Dai et al., 2019), creating a paradox of reduced iron and sustained FTH1 expression. This discrepancy raised the question of whether IRP–IRE-mediated control alone is sufficient to account for the observed increase in FTH1 expression, prompting us to explore alternative regulatory mechanisms.

Most human genes contain multiple polyadenylation sites (PASs), allowing cells to regulate gene expression by selecting different PASs in response to various signals (Tian et al., 2005). NUDT21, a critical component of the APA machinery, guides PAS selection during mRNA 3′-end processing, influencing cellular functions and fate decisions (Chen et al., 2024; Brumbaugh et al., 2018). In this study, we found that *Mtb* infection impairs NUDT21’s recognition of the *FTH1* 3′UTR (**Figure 4**) due to its dissociation from CPSF6 (**Figure 5**). We hypothesize that this NUDT21–CPSF6 dissociation may extend beyond *FTH1*, affecting the expression of other macrophage genes involved in the response to *Mtb*. Supporting this, NUDT21 knockdown significantly increased *Mtb* intracellular survival in BMDMs (**Figure 6**), highlighting its role in sustaining macrophage immunity. However, we did not assess whether this effect is FTH1-dependent. Given that *Mtb* exploits host iron metabolism for survival (Dai et al., 2023), further studies are needed to clarify the underlying mechanism.

Interestingly, our investigation of other genes involved in host–pathogen interactions, such as *IDO1*, revealed that APA reprogramming does not always favor the longer isoform. These gene-specific shifts in APA underscore the complexity of host gene regulation during *Mtb* infection and highlight the importance of APA in shaping the host’s immune response to the pathogen. Future studies should further explore changes in host transcript profiles during *Mtb* infection to identify additional APA-regulated genes, which could serve as molecular biomarkers or therapeutic targets.

Our study also demonstrated that *Mtb* infection impedes the recognition of UGUA motifs within the *FTH1* transcript in macrophages (**Figure 4**). Since UGUA motif are crucial for polyadenylation site selection (Yang et al., 2010), disruption of this interaction leads to the production of longer *FTH1* transcripts, which exhibit enhanced translational efficiency (**Figure 2**). This finding emphasizes the importance of cis-elements within the *FTH1* 3′UTR, and their corresponding trans-factors, in regulating gene expression during *Mtb* infection. Furthermore, we identified that the first 25 nucleotides in the DSE region influence polyadenylation site selection and protein expression in a fluorescent reporter system (**Figure 3C**, lane 5; 3D, lane 5). These results suggest that other cis-elements within the DSE, along with their corresponding trans-factors, contribute to the regulation of *FTH1* APA in *Mtb*-infected macrophages. While our study implicates these cis-elements, the potential role of trans-factors, such as CstF64, in the *Mtb*-induced *FTH1* APA shift remains speculative and warrants further investigation (Yao et al., 2012; Mukherjee et al., 2023).

Despite providing significant insights into the regulation of *FTH1* expression in *Mtb*-infected macrophages, our study has several limitations that should be considered. First, we used the avirulent H37Ra strain instead of the virulent H37Rv strain. H37Ra is commonly used in host-pathogen interaction studies due to its genetic similarity to H37Rv and its suitability for biosafety level 2 (BSL-2) laboratories. However, its attenuated virulence may lead to differences in macrophage responses and APA regulation. Future studies using virulent strains will be necessary to confirm whether the APA-mediated regulation of *FTH1* expression is conserved.

Additionally, while our study focuses on *Mtb* infection, *FTH1* upregulation has been observed in response to various intracellular pathogens and inflammatory conditions, suggesting a broader role in iron homeostasis and immune regulation. However, the extent and regulatory mechanisms of *FTH1* induction likely differ by pathogen and immune context. Further investigation is needed to delineate these mechanisms and their implications in different infectious contexts.

Furthermore, given the established role of CPSF6 in HIV-1 infection (Bejarano et al., 2019; Achuthan et al., 2018), the *Mtb*-induced release of CPSF6 from NUDT21 in co-infected individuals could potentially exacerbate HIV-1 replication, posing a public health concern, particularly in regions with high co-prevalence of both TB and HIV. While the clinical relevance of NUDT21–CPSF6 dysregulation in co-infection remains speculative, this pathway warrants investigation in cohorts with dual TB/HIV infections. We propose that targeting the NUDT21–CPSF6 axis could serve as a therapeutic strategy to enhance macrophage function and strengthen host defenses in these high-risk populations.

In summary, our study uncovers a novel APA mechanism that regulates *FTH1* expression in macrophages during *Mtb* infection. By disrupting the interaction between NUDT21 and CPSF6, *Mtb* shifts polyadenylation toward longer *FTH1* transcripts, leading to increased protein production, which ultimately compromises macrophage immunity. These findings emphasize the critical role of post-transcriptional regulation in host–pathogen interactions and open up new avenues for therapeutic strategies aimed at enhancing macrophage defenses against *Mtb*. Future research should further explore the roles of additional trans-factors in APA regulation and investigate potential approaches to modulate this pathway for better infection control.

## Data Availability

The datasets presented in this study can be found in online repositories. The names of the repository/repositories and accession number(s) can be found below: https://figshare.com/, https://doi.org/10.6084/m9.figshare.28428101.
